# Scandoside Exerts Anti-Inflammatory Effect Via Suppressing NF-κB and MAPK Signaling Pathways in LPS-Induced RAW 264.7 Macrophages

**DOI:** 10.3390/ijms19020457

**Published:** 2018-02-03

**Authors:** Jingyu He, Jiafeng Li, Han Liu, Zichao Yang, Fenghua Zhou, Ting Wei, Yaqian Dong, Hongjiao Xue, Lan Tang, Menghua Liu

**Affiliations:** 1 Bioengineering Research Centre, Guangzhou Institute of Advanced Technology, Chinese Academy of Sciences, Guangzhou 511458, China; jy.he@giat.ac.cn (J.H.); jiafengli2018@gmail.com (J.L.); ting.wei@siat.ac.cn (T.W.); 2 Guangdong Provincial Key Laboratory of New Drug Screening, School of Pharmaceutical Sciences, Southern Medical University, Guangzhou 510515, China; hanliuchn@outlook.com (H.L.); zcyangchn@163.com (Z.Y.); yqdongchn@163.com (Y.D.); xuehongjiao@sina.com (H.X.); tl405@smu.edu.cn (L.T.); 3 School of Traditional Chinese Medicine, Southern Medical University, Guangzhou 510515, China; wendy515@fimmu.com

**Keywords:** scandoside, nuclear transcription factor kappa-B alpaha, mitogen-activated protein kinase, anti-inflammation

## Abstract

The iridoids of *Hedyotis diffusa* Willd play an important role in the anti-inflammatory process, but the specific iridoid with anti-inflammatory effect and its mechanism has not be thoroughly studied. An iridoid compound named scandoside (SCA) was isolated from *H. diffusa* and its anti-inflammatory effect was investigated in lipopolysaccharide (LPS)-induced RAW 264.7 macrophages. Its anti-inflammatory mechanism was confirmed by in intro experiments and molecular docking analyses. As results, SCA significantly decreased the productions of nitric oxide (NO), prostaglandin E_2_ (PGE_2_), tumor necrosis factor-α (TNF-α) and interleukin-6 (IL-6) and inhibited the levels of inducible nitric oxide synthase (iNOS), cyclooxygenase-2 (COX-2), TNF-α and IL-6 messenger RNA (mRNA) expression in LPS-induced RAW 264.7 macrophages. SCA treatment suppressed the phosphorylation of inhibitor of nuclear transcription factor kappa-B alpaha (IκB-α), p38, extracellular signal-regulated kinase (ERK) and c-Jun N-terminal kinase (JNK). The docking data suggested that SCA had great binding abilities to COX-2, iNOS and IκB. Taken together, the results indicated that the anti-inflammatory effect of SCA is due to inhibition of pro-inflammatory cytokines and mediators via suppressing the nuclear transcription factor kappa-B (NF-κB) and mitogen-activated protein kinase (MAPK) signaling pathways, which provided useful information for its application and development.

## 1. Introduction

Inflammation is a natural defense response when body is invaded by bacteria, viruses and fungi [1]. Lipopolysaccharide (LPS) that is a common endotoxin derived from the outer membrane of gram-negative bacterial can cause a series of inflammatory reactions [2,3]. During the inflammatory process, macrophages can be recruited to inflammatory sites and plays an essential role by various signals that stimulate intracellular cascades [4]. Consequently, inflammatory cytokines such as tumor necrosis factor-α (TNF-α) and interleukin-6 (IL-6), and inflammatory mediators such as nitric oxide (NO) and prostaglandin E_2_ (PGE_2_) were increased [5,6,7]. Nuclear factor-Kappa B (NF-κB) and mitogen-activated protein kinases (MAPKs) are two crucial pathways to regulate the transcription of inflammatory cytokines and mediators during inflammatory process activated by LPS stimulation [5,8,9]. Once activated, NF-κB and MAPKs can induce collaboratively the expression of pro-inflammatory cytokine genes and the release of cytokines under the inflammatory process [10]. Therefore, molecule or chemical targeting of NF-κB and/or MAPK signaling pathways are considered to be a potential anti-inflammatory agent, which is a strategy for the treatment of inflammation-related disorders.

**Figure 1:**
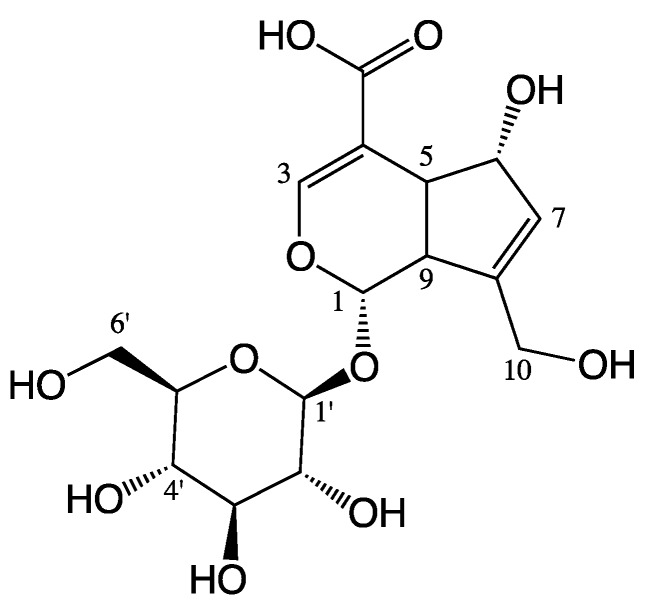
Chemical structure of scandoside (SCA).

*Hedyotis diffusa* Willd as a famous traditional Chinese medicine is widely used in the South of China and other Asian countries [11]. Traditionally, it is used for the treatment of bronchitis, arthritis, rheumatism, urethral infection, appendicitis, sore throat, contusions, ulcerations and malignancies [12]. Modern pharmacological studies have proven that *H. diffusa* has multiple effects, such as anti-inflammatory, anti-cancer, immune-modulating, neuroprotective and hepatoprotective activities [13]. It was found that the iridoid compounds possibly responsible for the anti-inflammatory effect of *H. diffusa* [14,15]. In our previous study, *H. diffusa* treatment could significantly decrease the pro-inflammatory cytokines and mediators in LPS-induced renal inflammation mice and thus alleviate the inflammatory reaction. Furthermore, ten iridoids were detected in serum of the extract treated group, indicating iridoids might be responsible for the therapeutic effect of *H. diffusa* [16]. However, the specific iridoid with anti-inflammatory effect and its anti-inflammatory mechanism are still unclear. In this study, an iridoid compound named scandoside (SCA) (Figure 1; PubChem CID: 21602023) was isolated from *H. diffusa* and its anti-inflammatory effect was investigated using LPS-induced RAW 264.7 macrophages. Moreover, its anti-inflammatory mechanisms were further clarified.

## 2. Results

### 2.1. Effects of SCA on RAW 264.7 Macrophage Viability

**Figure 2:**
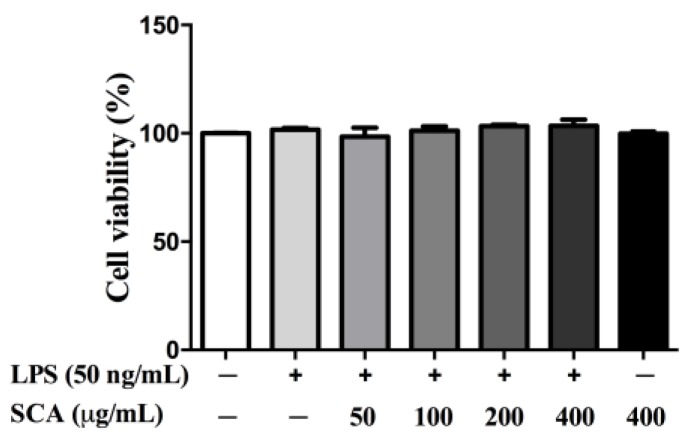
Effect of SCA on the viability of RAW 264.7 macrophages. RAW 264.7 macrophages were treated with SCA at the concentrations of 0, 50, 100, 200 and 400 μg/mL, respectively, for 1 h, and then stimulated with 50 ng/mL lipopolysaccharide (LPS) for 24 h. RAW 264.7 macrophages were also treated with SCA at the concentration of 400 μg/mL without LPS for 24 h. Cell viability was detected by cell counting kit-8 (CCK-8) assay.

The cytotoxicity of SCA on RAW 264.7 macrophages was examined by the cell counting kit-8 (CCK-8) assay. As shown in Figure 2, the percentages of cell viabilities were from 98.38% to 103.48%. Cell viabilities were not significantly affected by various concentrations of SCA after a 24-h treatment in the presence of 50 ng/mL LPS. RAW 264.7 macrophages were also treated with SCA at the concentration of 400 μg/mL without LPS for 24 h, indicating that SCA was non-toxic to RAW 264.7 macrophages below 400 μg/mL.

### 2.2. Effects of SCA on Inflammatory Mediators and Inflammatory Cytokines in RAW 264.7 Macrophages

**Figure 3:**
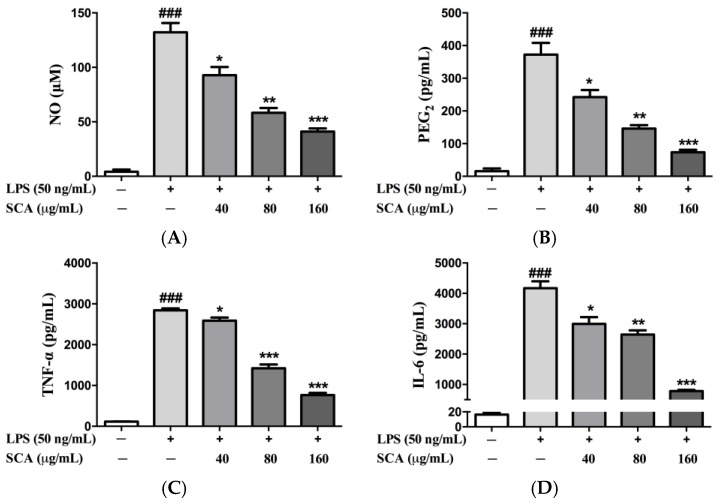
Effects of SCA on the productions of NO (**A**); PGE_2_ (**B**); TNF-α (**C**) and IL-6 (**D**). RAW 264.7 macrophages were treated with SCA at the concentrations of 40, 80 and 160 μg/mL, respectively, for 1 h, and then stimulated with 50 ng/mL LPS for 24 h. The concentrations in the cell-free culture were measured by enzyme-linked immunosorbent assay (ELISA). * *p* < 0.05, ** *p* < 0.01 and *** *p* < 0.001 versus LPS-only treatment group; ^###^ *p* < 0.001 versus control group.

As shown in Figure 3, the significant increases of inflammatory mediators (NO and PEG_2_) and inflammatory cytokines (TNF-α and IL-6) in the LPS-treatment group were observed when compared with the control group. Conversely, SCA treatment groups gave different behaviors. SCA treatment significantly reduced the productions of NO, PEG_2_, TNF-α and IL-6 (*p* < 0.05) in concentration-dependent manners.

### 2.3. Effects of SCA on TNF-α and IL-6 Messeger RNA (mRNA) Expression in LPS-Induced RAW 264.7 Macrophages

**Figure 4:**
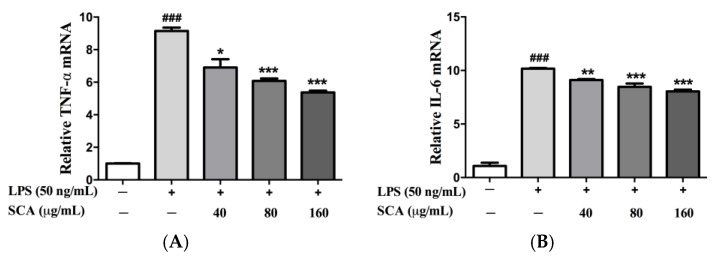
Effects of SCA on TNF-α (**A**) and IL-6 (**B**) mRNA expressions. RAW 264.7 macrophages were treated with SCA (40, 80 and 160 μg/mL) for 1 h and then stimulated with LPS (50 ng/mL) for 24 h. The TNF-α and IL-6 mRNA expressions were analyzed by real-time PCR. * *p* < 0.05, ** *p* < 0.01 and *** *p* < 0.001 versus LPS-only treatment group; ^###^ *p* < 0.001 versus control group.

The mRNA expression of TNF-α and IL-6 were investigated to find out whether SCA could regulate their transcriptional levels. As shown in Figure 4, SCA treatment could significantly down-regulate the mRNA levels of TNF-α and IL-6 compared with the LPS-treated group. The reduced mRNA levels of TNF-α and IL-6 were roughly consistent with their protein levels when treated with SCA.

### 2.4. Effects of SCA on iNOS and COX-2 Proteins and mRNA Expressions in LPS-Induced RAW 264.7 Macrophages

**Figure 5:**
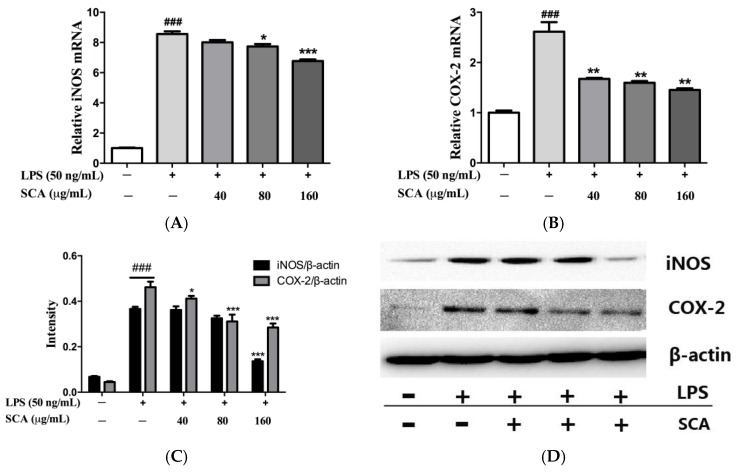
Effects of SCA on iNOS and COX-2 mRNA (**A** and **B**) and protein expressions (**C** and **D**). RAW 264.7 macrophages were treated with SCA (40, 80 and 160 μg/mL) for 1 h and then stimulated with LPS (50 ng/mL) for 24 h. The iNOS and COX-2 mRNA expressions were analyzed by RT-PCR. The iNOS and COX-2 proteins were analyzed by Western blotting. The bar chart shows the quantitative evaluation of iNOS and COX-2 protein bands by densitometry. * *p* < 0.05, ** *p* < 0.01 and *** *p* < 0.001 versus LPS-only treatment group; ^###^ *p* < 0.001 versus control group.

To study the issue of whether SCA suppressed the production of NO and PGE_2_ via inhibiting the expression of their corresponding synthases (iNOS and COX-2), the protein and mRNA expression of iNOS and COX-2 were measured, respectively. The results of real-time PCR (RT-PCR) analyses showed that LPS induced the significant up-regulation of the mRNA transcript levels of iNOS and COX-2. The SCA-treated group could significantly down-regulate the transcriptional levels of iNOS and COX-2 mRNA compared with the only LPS-treated group, in a concentration-dependent manner (Figure 5A,B). SCA treatment, concentration-dependently, reduced the protein levels of iNOS and COX-2 (Figure 5C,D). The reduction of iNOS and COX-2 mRNA and protein levels were consistent with the reduced production of NO and PGE_2_, respectively.

### 2.5. Effects of SCA on NF-κB and MAPK Signaling Pathways in LPS-Induced RAW 264.7 Macrophages

**Figure 6:**
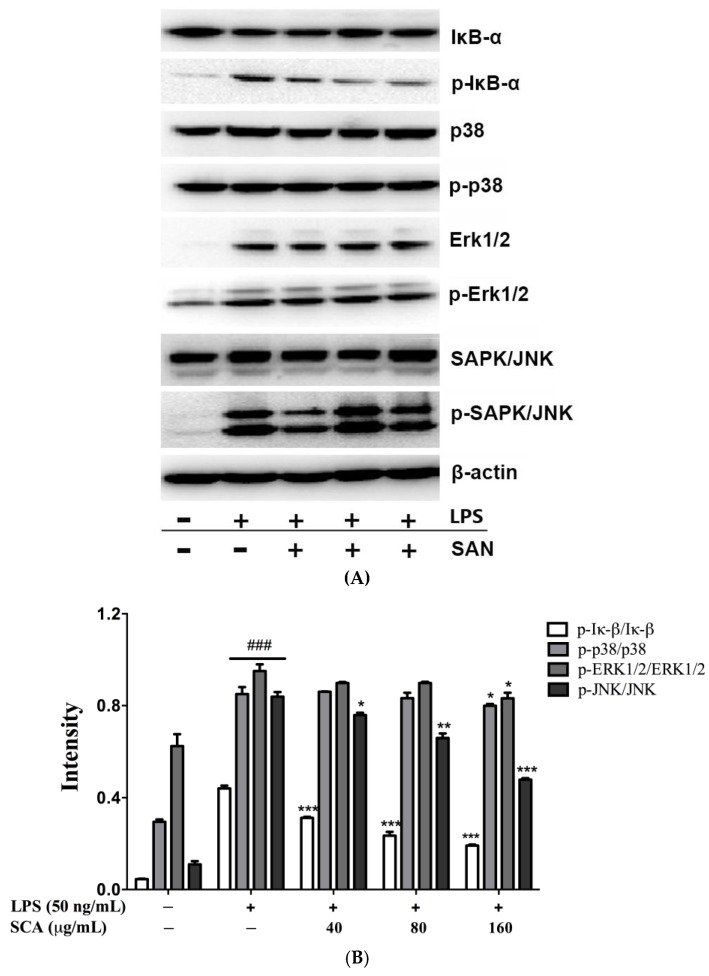
Effect of SCA on IκBα, p38, extracellular signal-regulated kinase (ERK)1/2 and c-Jun N-terminal kinase (JNK)phosphorylation. (**A**) RAW 264.7 macrophages were treated with SCA (40, 80 and 160 μg/mL) for 1 h and then stimulated with LPS (50 ng/mL) for 24 h. IκBα, p38, ERK1/2 and JNK, alone with their phosphorylated products, were analyzed by Western blotting. β-actin was used as the internal control for the lysate and cytosolic fraction. p-IκBα, p-p38, p-ERK1/2 and p-JNK were normalized with IκBα, p38, ERK1/2 and JNK, respectively; (**B**) The bar chart shows the quantitative evaluation of protein bands by densitometry * *p* < 0.05, ** *p* < 0.01 and *** *p* < 0.001 versus LPS-only treatment group; ^###^ *p* < 0.001 versus control group.

To study the potential mechanism of anti-inflammation, the effects of SCA on IκB-α phosphorylation and degradation were investigated in LPS-stimulated RAW 264.7 macrophages. As shown in Figure 6, LPS-induced IκB-α phosphorylation was significantly decreased after SCA treatment in a concentration-dependent manner. We also examined to the regulatory effect of SCA on the MAPK signaling pathway. As results, p38, Erk1/2, and JNK in RAW 264.7 macrophages stimulated by LPS were highly phosphorylated. JNK phosphorylation was inhibited significantly by SCA in a concentration-dependent manner. For the effect on MAPK, SCA treatment remarkably decreased p38 and ERK1/2 phosphorylation at the highest concentration level (160 μg/mL).

### 2.6. Molecular Docking Analysis

**Figure 7:**
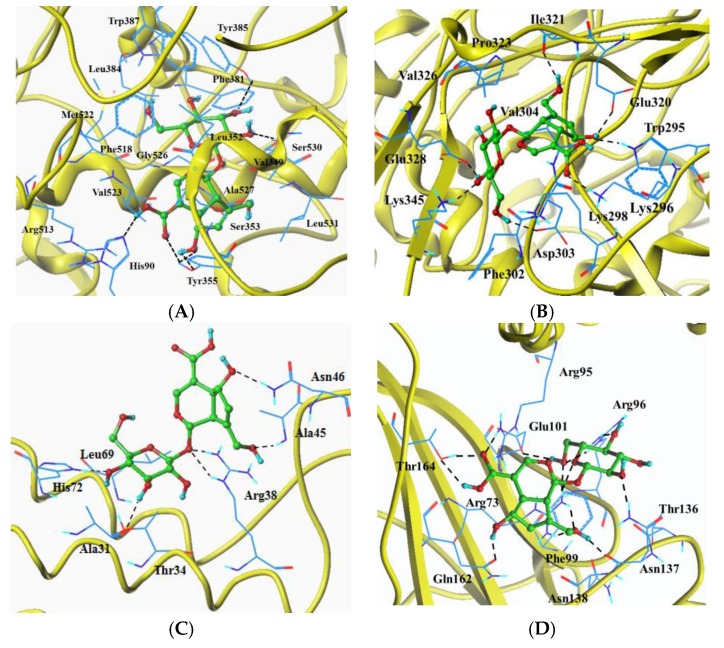
The best docked pose of SCA with target proteins. Docked orientation of SCA (colored in green) with corresponding secondary protein structure (colored in yellow) and amino acid residues (colored in blue) of COX-2 (1CX2) (**A**); iNOS (4NOS) (**B**); PEG_2_ (4AL0) (**C**) and IκB (1NFI) (**D**).

The docking results of SCA with target proteins (iNOS, COX-2, PEG_2_ and IκB) are shown in Figure 7 and Table 1. Total scores of complexes of SCA with iNOS, COX-2 and IκB were close, but the obtained score of SCA with PEG_2_ was much lower. In docking experiments on COX-2, His90, Tyr355, Tyr385 and Ser530 formed hydrogen bonds. Sixteen hydrophobic interaction binding residues His90, Val349, Leu352, Ser353, Tyr355, Phe381, Leu384, Tyr385, Trp387, Phe518, Met522, Val523, Gly526, Ala527, Ser530 and Leu531 were found. For the docking experiments on iNOS, amino acid residues Trp295, Lys296, Asp303, Glu320, Ile321, Glu328 and Lys345 formed hydrogen bonds. Twelve common amino acid residues with hydrophobic interaction were Trp295, Lys296, Lys298, Phe302, Asp303, Val304, Glu320, Ile321, Pro323, Val326, Glu328 and Lys345. As for PEG_2_, SCA could bind to the site Ala31, Arg38, Ala45, Asn46 and His72 of the crystal structure by hydrogen bonds. Besides, the hydrophobic interactions were found in the sites Thr34 and Leu69 of the complexes. Amino acid residues Arg73, Arg95, Arg96, Glu101, Asn137, Gln162 and Thr164 in the crystal structure of IκB formed hydrogen bonds with SCA. Together with these amino acid residues, the other residues Phe99, Thr136 and Asn138 had hydrogen interactions in the IκB-ligand complexes.

## 3. Discussion

A series of studies have proven that the extracts of fruits, vegetables, herbal plants and their formulas show great anti-inflammatory effects [17,18,19,20]. As known, phytochemicals are responsible for these bioactivities. Thus, it is necessary to identify which phytochemical in the natural resources contributes to the anti-inflammatory effect. Various phytochemicals, especially flavonoids and terpenoids, have been found to be anti-inflammatory compounds and their anti-inflammatory mechanisms have also been clarified [21,22,23,24,25,26]. Among them, the iridoids attracted much attention and some iridoid compounds have been proven to be of anti-inflammatory [27,28]. In this study, SCA, an iridoid compound, was successfully isolated from *H. diffusa* which is usually used for the treatment of inflammation in the clinic. SCA is not a main compound and is of low content in *H. diffusa*, but it could be absorbed in blood and bind to inflammatory tissues [16,29,30]. To elucidate the anti-inflammatory effect of SCA, the pro-inflammatory cytokines and mediators in LPS-induced RAW 264.7 macrophages were assayed. As results, SCA showed an anti-inflammatory effect due to the significant reductions of NO, PGE_2_, TNF-α and IL-6 after treatment.

The productions of inflammation-related factors are regarded as the indicators for the inflammation reaction. Pro-inflammatory cytokines such as TNF-α and IL-6 play crucial roles in the development of inflammatory diseases and are involved in immunity and autoimmune diseases [31,32]. TNF-α and IL-6 mRNA expression in LPS-stimulated RAW 264.7 macrophages were measured. The results indicated that SCA could significantly suppress TNF-α and IL-6 mRNA expression. NO predominately produced by iNOS is a crucial indicator of inflammation. PGE_2_ synthesized by COX-2, is an important mediator due to the various biological effect associated with inflammation [5,6,7]. In RAW 264.7 macrophages, LPS effectively activated iNOS and COX-2 transcription leading to the overproductions of NO and PEG_2_ [33]. The results demonstrated that SCA significantly decreased the productions of NO and PEG_2_ by suppressing iNOS and COX-2, respectively, and at the same time markedly inhibited iNOS and COX-2 mRNA expression.

NF-κB is a key signaling pathway related to regulating the transcription of numerous pro-inflammatory cytokines and mediators, including TNF-α, IL-6, iNOS and COX-2 [5,8]. The phosphorylation of IκBα by IKKα is a critical process for the activation of NF-κB. SCA was able to significantly inhibit NF-κB activation via decreasing the phosphorylation of IκB. MAPKs, including ERK, JNK and p38 which also take part in the expression regulation of inflammation-related genes, leading to the overproduction of pro-inflammatory cytokines [34,35]. SCA significantly inhibited LPS-induced phosphorylation of p38, ERK and JNK in Raw 264.7 macrophages, hence displaying its anti-inflammatory effects and mechanism. It is reported that the NF-κB and MAPK signaling pathways can collaborate synergistically to promote the expression and release target genes. As the results show, SCA exerted the anti-inflammatory effect via suppressing NF-κB and MAPK signaling pathways.

The anti-inflammatory property of SCA was also confirmed by molecular docking analysis. SCA binds to the COX-2 active sites (His90, Tyr355 Tyr385 Trp387, Met522, Val523 and Ser530) to form a complex, which was consistent with that of anti-inflammatory compounds [36]. Similarly, SCA could bind to the key amino acids of iNOS (Lys296 and Glu320), PEG_2_ (His72) and IκB (Arg95) when forming enzyme–ligand complexs [26]. These findings supported that SCA showed anti-inflammatory effects by affecting the activities of these proteins. These results also indicated that SCA was responsible for the anti-inflammatory effect of *H. diffusa*; SCA should be considered as a crucial marker for quality control, pharmacokinetics and drug development of herbaceous plants. Importantly, the other herbaceous plants containing SCA should be paid attention regarding inflammatory effects. In view of the experimental results achieved in vitro, further in vivo studies of the anti-inflammatory effect of SCA are needed.

## 4. Materials and Methods

### 4.1. Plant, Chemicals and Reagents

The materials of *H. diffusa* was purchased from Bozhou Chinese Medicine Processing Plant (Bozhou, Anhui, China) and identified by Doctor Jing Wang (School of Traditional Chinese Medicine, Southern Medical University, Guangzhou, China).

RAW 264.7 murine macrophages were purchased from Cell Bank of the Chinese Academy of Science (Shanghai, China). SCA, which was only used as the reference compound in the isolation and identification process, was purchased from Shanghai yuanye Bio-Technology Co., Ltd (Shanghai, China). Dulbecco’s modification of Eagle’s medium (DMEM, No. 12430-054) and fetal bovine serum (FBS, No. 10099141) were purchased from Gibco (Thermo Scientific, Waltham, MA, USA). LPS obtained from *Escherichia coli* O111:B4, was purchased from Sigma-Aldrich Co. LLC. (St. Louis, MO, USA). The antibodies of iNOS), COX-2, IκB-α, p38 and ERK1/2 were purchased from Proteintech Group, Inc. (Chicago, IL, USA). The antibodies of p-IκB-α, p-p38, p-ERK1/2, JNK and p-JNK were purchased from Cell Signaling Technology, Inc. (Danvers, MA, USA). CCK-8 was purchased from Dojindo (Kumamoto, Japan). The ELISA kits of IL-6, IL-β1 and TNF-α were purchased from Neobioscience Technology Company (Shenzhen, China). NO ELISA kit was purchased from Beyotime Biotechnology (Shanghai, China), and PEG_2_ was purchased from Enzo Life Sciences (New York, NY, USA). All other reagents were of analytical grade.

### 4.2. Sample Preparation

The dried and ground *H. diffusa* (5.0 kg) were extracted with 50 L of 75% ethanol (*v/v*) at 80 °C for 1 h. The extraction was repeated once after flirtation. The filtrate was evaporated under vacuum (Tokyo Rikakikai Co., Ltd., Tokyo, Japan) at 60 °C to yield the concentrate with the relative density of 1.15 g/mL. Adding 0.5% active carbon powder to the concentrate, the supernatant was obtained by centrifugation at 5000 rpm and then dried in vacuum. The extract was submitted to a macroporous resin column (9 cm i.d. × 45 cm), and eluted with water (10 L). Subsequently, 40 fractions (250 mL per fraction) were obtained after elution with 30% methanol (10 L, *v/v*). Using commercial reference SCA, the fraction containing SCA was confirmed by HPLC-DAD (Waters 1525; Waters Corp., Milford, MA, USA). Fractions 26, 27 and 28 were combined and evaporated in vacuum at 60 °C to give the residue (0.85 g). The residue was dissolved in 30% methanol (*v/v*), and then purified by semi-preparative HPLC (Waters Delta 600; Waters Corp., Milford, MA, USA) to afford SCA (4.9 mg) under the condition (column: YMC-Pack ODS-A (20 mm i.d. × 250 mm, 5 μm; YMC Co., Ltd., Kyoto, Japan); solvent: MeOH/water solution, 30:70–75:25 *v/v*, linear gradient in 50 min; flow rate: 10 mL/min; Detection wavelength: 254 nm). SCA was identified by comparison of the spectral data of HRMS (Triple TOF^TM^ 5600 plus; AB SCIEX, Foster City, CA, USA) and ^1^H NMR (Bruker AVANCE ARX-300; Bruker Corp., Swiss).

Scandoside: white powder; ^1^H NMR (300 MHz, CD_3_OD): δ 7.37 (1H, s, H-3), 5.65 (1H, s, H-7), 5.06 (1H, brd, *J* = 6.6 Hz, H-1), 4.73 (1H, brd, H-1’), 4.47 (1H, brd, H-6), 4.39 (1H, d, *J* = 15.0 Hz, H-10a), 4.09 (1H, d, *J* = 15.0 Hz, H-10b), 2.96 (1H, m, H-9), 2.88 (1H, m, H-5); [M+H]^+^ calculated for C_16_H_23_O_11_, 391.12349; found, 391.12307 [37,38]. The purity of SCA was confirmed by HPLC-DAD to be over 97%.

### 4.3. Cell Line and Culture

RAW 264.7 macrophages were cultured in DMEM supplemented with 10% FBS, 100 units/mL penicillin and 100 μg/mL streptomycin and maintained in a Thermo carbon dioxide incubator (Thermo Fisher Scientific, Waltham, MA, USA) with a humidified atmosphere of 95% air and 5% CO_2_ at 37 °C. In the study, the cell model was established according to the reference [27] and the results of cell viability assay. The inflammatory cell model was incubated with 50 ng/mL LPS for 24 h after being treated with SCA for 1 h.

### 4.4. CCK-8 Assay for Cell Viability Evaluation

RAW 264.7 macrophages were seeded in 96-well plates at a density of 1 × 10^4^ cells/well and incubated for 24 h. The cells were treated with SCA at the concentrations of 0, 25, 50, 100, 200 and 400 μg/mL for 1 h and subsequently stimulated with 50 ng/mL LPS for 24 h. Finally, 10 μL of CCK-8 were added in each plates, incubated for 1 h at 37 °C and then determined with the absorption wavelength at 450 nm using TECAN Microplate Reader (Tecan Group Ltd., Männedorf, Switzerland). The cell viability was measured by comparing the absorbance values of treatment groups with those of the control group.

### 4.5. ELISA Assay of NO, PGE_2_, TNF-α and IL-6

The concentrations of NO, PGE_2_, TNF-α and IL-6 were measured according to the manufacture’s instruction of commercial ELISA kits. Briefly, RAW 264.7 macrophages were seeded in 96-well plates at a density of 1 × 10^4^ cells/well and incubated for 24 h. Then, the cells were treated with SCA (40, 80 and 160 μg/mL) for 1 h. After they were stimulated with 50 ng/mL LPS for 24 h, the cell supernatants were collected for the determination of NO, PGE_2_, TNF-α and IL-6 levels by TECAN Microplate Reader. The absorption wavelengths were 540, 450, 450 and 450 nm for NO, PGE_2_, TNF-α and IL-6, respectively.

### 4.6. RT-PCR Assay

RAW 264.7 macrophages were seeded in 6-well plates at a density of 2 × 10^5^ cells/well and incubated at 37 °C for 24 h. A series of concentrations of SCA (40, 80 and 160 μg/mL) were used to treat the cells for 1 h and stimulated with LPS (50 ng/mL) for 24 h. Total RNA was extracted from these cells using RNAprep pure cell kit (Qiagen, Valencia, CA, USA). The RNA purity and content were determined by measuring the absorbance ratio at 260/280 nm. Subsequently, the total RNA was converted into cDNA with a reverse transcription system containing 4 μL 5× prime Script RT Master MIX (perfect Real Time), 0.5 μg total RNA, and 15.5 μL RNase-free water. The cDNA was used for RT-PCR by an Applied Biosystems^®^ 7500 Fast Real-time PCR System (Thermo Fisher Scientific, Waltham, MA, USA) for analysis of iNOS, COX-2, TNF-α and IL-6. The RT-PCR reaction system contained 10 μL SYBR Premix EX Taq(2×), 1 μL forward primer (10 µM), 1 μL reverse primer (10 µM) and 8 μL cDNA under the reaction conditions: 50.0 °C for 3 min, 95.0 °C for 3 min, followed by 40 cycles for 95.0 °C for 10 s and 60.0 °C for 30 s. The primers for iNOS, COX-2, TNF-α and IL-6 were used as shown in Table 2.

### 4.7. Western Blot Analysis

RAW 264.7 macrophages were seeded in 6-well plates at a density of 2 × 10^5^ cells/well, after being incubated at 37 °C for 24 h, the cells were treated with SCA (40, 80 and 160 μg/mL) for 1 h and then stimulated with LPS (50 ng/mL) for 24 h. Subsequently, the cells were collected for protein analysis. One hundred microlitre of cell lysis buffer (10 mM Tris-HCl, 0.15 M NaCl, 5 mM ethylenediaminetetraacetic acid (EDTA), 1% Triton × 100, 5 mM dithiothreitol (DTT) and 0.1 mM phenylmethanesulfonyl fluoride (PMSF)) were added and incubated for 30 min at 4 °C, and then centrifuged at 12,000 rpm for 10 min. After collecting the supernatant in a new tube, the protein concentration was measured by BCA Protein Assay Kit (Beyotime Biotechnology, Shanghai, China). All protein samples were loaded onto 12% sodium dodecyl sulfate-polyacrylamide gel electrophoresis (SDS-PAGE). The immunoblot was blocked for 2 h with 10% nonfat milk at room temperature, and then incubated overnight with the primary antibodies, including iNOS (1:1000), COX-2 (1:1000), IκB-α (1:1000), p-IκB-α (1:1000), p38 (1:1000), p-p38 (1:1000), ERK1/2 (1:1000), p-ERK1/2 (1:1000), JNK (1:1000), p-JNK (1:1000) and β-actin (1:1000), at 4 °C. After being washed for three times, the membranes were incubated with the secondary antibody (1:10000) for 1 h at room temperature. The blots were detected by ECL Chemiluminescence method plus Western Blotting Detection System (FluorChem R, ProteinSimple, San Jose, CA, USA).

### 4.8. Molecular Docking

Docking experiments were performed using Sybyl.v 7.3 (Tripos, Inc., St. Louis, MA, USA). Chemical structure of SCA was downloaded from Pubmed compound database (Available online: https://www.ncbi.nlm.nih.gov/pccompound/). Energy minimization was performed using the Tripos force field and Gasteiger–Huckel charges and the conjugate algorithm with a convergence criterion of 0.001 kcal/(mol A). X-ray crystal structures of target proteins of iNOS (PDB ID: 4NOS), COX-2 (PDB ID: 1CX2), PEG_2_ (PDB ID: 4AL0) and IκB (PDB ID: 1NFI) were retrieved from the RCSB Protein Data Bank (Available online: http://www.rcsb.org/pdb/home/home.do) and arranged using Sybyl.v 7.3 software suite. The protomol was generated using ligand mode for COX-2 by extracting SC-558 from the initial COX-2 crystal structure occupying the binding site [36]. While the protomols for iNOS, PEG_2_ and IκB were generated using residue mode, based on the active residues reported [26]. Surflex-dock Program interfaced with Sybyl.v 7.3 was used in docking and both hydrogen and heavy atoms were chosen in result optimization.

### 4.9. Statistical Analyses

All experiments were performed in triplicate. Data were analyzed by Graphpad prism (Graphpad Software, San Diego, CA, USA) and presented as mean ± standard deviation (SD). One-way ANOVA followed by Student’s *t* test was used to analyze the statistical differences between groups. *p* < 0.05 was considered as a significant difference.

## 5. Conclusions

SCA significantly decreased the productions of NO, PGE_2_, TNF-α and IL-6 and inhibited the levels of iNOS, COX-2, TNF-α and IL-6 mRNA expression in LPS-induced RAW 264.7 macrophages. Its anti-inflammatory effect is associated with suppressing the NF-κB and MAPK signaling pathways.

## Figures and Tables

**Table 1:** Virtual binding score values and interaction between the ligand and amino acid residues in scandoside protein–ligand complexes.

Target Protein	Total Score ^1^	Crash ^2^	Polar ^3^	Hydrogen Bonds	Eletrostatic Interaction	Hydrophobic Interaction (0.5 Å)
COX-2(1CX2)	9.0084	−3.0357	2.9740	His90, Tyr355(3), Tyr385, Ser530	His90, Arg513	His90, Val349, Leu352, Ser353, Tyr355, Phe381, Leu384, Tyr385, Trp387, Phe518, Met522, Val523, Gly526, Ala527, Ser530, Leu531
iNOS(4NOS)	9.2757	−1.4618	7.6190	Trp295, Lys296, Asp303, Glu320, Ile321, Glu328, Lys345	Lys296	Trp295, Lys296, Lys298, Phe302, Asp303, Val304, Glu320, Ile321, Pro323, Val326, Glu328, Lys345
PEG_2_(4AL0)	6.2647	−1.2700	5.0136	Ala31, Arg38(2), Ala45, Asn46, His72		Ala31, Thr34, Arg38, Ala45, Asn46, Leu69, His72
IκB(1NFI)	9.0953	−0.6933	9.9542	Arg73(2), ^a^ Arg95, Arg96(2), Glu101, Asn137(2), Gln162, Thr164(2)	^a^ Arg95	Arg73, ^a^ Arg95, Arg96, Phe99, Glu101, Thr136, Asn137, Asn138, Gln162, Thr164

^1^ Total score: total docking score expressed in –log (Kd) units to represent binding affinities. ^2^ Crash: the degree of inappropriate penetration by the ligand into the protein and of interpenetration between ligand atoms that are separated by rotatable bonds. ^3^ Polar: the effect of polar non-hydrogen bonding interaction to the total score. The number behind some residues stands for the number of hydrogen bond between the residue and the ligand. ^a^ Residues located in E subunit of IκB (1NFI) and other residues locate in A subunit.

**Table 2:** The primers used for RT-PCR analysis.

Cytokines	Sense Primer Sequence5’-3	Antisense Primer Sequence5’-3
TNF-α	GCGACGTGGAACTGGCAGAA	CAGTAGACAGAAGAGCGTGGTG
IL-6	GTTGCCTTCTTGGGACTGAT	CATTTCCACGATTTCCCAGA
iNOS	TGGAGCGAGTTGTGGATTGT	CTCTGCCTATCCGTCTCGTC
COX-2	ACCTGGTGAACTACGACTGC	TGGTCGGTTTGATGTTACTG
β-actin	TGCTGTCCCTGTATGCCTCTG	GCTGTAGCCACGCTCGGTCA
